# Study of burnout and depressive symptoms in doctors at a central level, state hospital

**DOI:** 10.4102/sajpsychiatry.v29i0.1866

**Published:** 2023-02-28

**Authors:** Ariefdien Nazeema, Karishma Lowton, Zenaida Tenea, Ani Anic, Preethi Jayrajh

**Affiliations:** 1 Department of Psychiatry, Faculty of Health Sciences, University of the Witwaterstrand, Johannesburg, South Africa

**Keywords:** burnout, depression, doctors, Maslach, Charlotte Maxeke Johannesburg Academic Hospital

## Abstract

**Background:**

Doctors are at high risk of burnout, which has far-reaching consequences on an individual and organisational level. Several studies have shown an association between burnout and depression.

**Aim:**

This study aimed to determine the rate of burnout and depressive symptoms among doctors, as well as factors associated with both conditions.

**Setting:**

Charlotte Maxeke Johannesburg Academic Hospital.

**Methods:**

Burnout was measured using the Maslach Burnout Inventory–Human Services Survey and defined as the total score of high emotional exhaustion (≥ 27 points) + high depersonalisation (≥ 13 points). Individual subscales were analysed separately. Depressive symptoms were screened using the Patient-Health Questionnaire-9 (PHQ-9) and a score of ≥ 8 was deemed indicative of depression.

**Results:**

Of the respondents (*n* = 327 for burnout and *n* = 335 for depression), 46.2% screened positive for burnout, whilst 53.73% screened positive for depression. Factors associated with increased burnout risk were younger age; Caucasian race; internship and/or registrarship; the discipline of emergency medicine; and having a prior psychiatric diagnosis of depressive and/or anxiety disorder. Factors associated with increased risk of depressive symptoms were females; younger age; being an intern, medical officer or registrar; disciplines of anaesthetics and obstetrics and gynaecology; having a prior psychiatric diagnosis of depressive and/or anxiety disorder; and family history of psychiatric disorder.

**Conclusion:**

A high rate of burnout and depressive symptoms was determined. Although there is an overlap between the two conditions in terms of both symptomatology and risk factors, specific risk factors were determined for each in this population.

**Contribution:**

This study highlighted the rate of burnout and depressive symptoms experienced by doctors at the state level hospital necessitating individual and institutional interventions to address this.

## Introduction

The term ‘burnout’, first used by Herbert Freudenberger in 1974,^1^ described strain arising from prolonged exposure to chronic, job-related stress. In addition, Maslach and Schaufeli supported this in their findings.^2^

Maslach and Jackson^3^ redefined the concept as a syndrome consisting of three dimensions:

- High emotional exhaustion (EE) – chronic fatigue and low motivation
- High depersonalisation (DP) – a feeling of distance from one’s job, cynicism and negativism
- Low personal accomplishment (PA)

Further research by Maslach led to the development of the Maslach Burnout Inventory–Human Services Survey (MBI–HSS), which is considered the gold standard measure for assessing burnout.^4^ Healthcare professionals are considered vulnerable to burnout with rates varying between 21% and 100% amongst South African doctors.^5,6,7,8,9,10,11^ Internationally, the 2019 Medscape National Physician Burnout, Depression and Suicide Report quotes burnout prevalence rates of 44%^12^ especially in disciplines of urology, neurology, internal medicine, surgery and anaesthetics. Similarly, a European-based systematic review found that certain surgical specialities and oncology personnel were more likely to experience burnout.^13^ Bureaucratic tasks, followed by long working hours, were the largest contributors resulting in higher dissatisfaction at work.^14^

Many studies explored the risk factors contributing to burnout, which may be divided into three domains:^13,14,15,16,17^

- *Occupational factors* – discipline, job position (registrarship in particular), clerical work, working hours, high patient load and risk of litigation
- *Physicians’ attributes* – younger age, female sex, certain personality traits (e.g., neuroticism) and maladaptive coping skills
- *Organisational factors* – poor leadership, hostile work environment and lack of peer support

The consequences of burnout can generally be divided into four domains:^13,18,19,20^

- *Physical consequences* – coronary heart disease, chronic fatigue and increased risk of early mortality
- *Psychological consequences* – depressive symptoms, substance abuse and increased risk of suicide
- *Professional consequences* – absenteeism, medical errors, less productivity and resignations
- *Organisational consequences* – financial losses incurred in training and recruitment because of clinicians leaving the organisation, as well as malpractice costs

Burnout is often associated with depression with considerable overlap of symptoms^20^; however, burnout is directly linked to work-related stressors, whereas depression may occur in the absence of work stress.^19^ As a result of the overlap, correlations between measures of the two constructs^16^ may be artificially inflated. The overlap may be more profound and reflect burnout as a depressive condition.^21^ According to most researchers, burnout and depression are independent entities, differentiated by symptoms specific to depression, such as anhedonia, inappropriate guilt and definitive mood change.^22^

An international systematic review estimated the prevalence of depressive symptoms among registrars as 28.8%^20^ compared with South African rates of 30% – 40.7%.^21,22,23^ According to the American Foundation for Suicide Prevention, training physicians are at an increased risk of suicide, with a fourfold increased risk of suicidal ideation during the first three months of internship.^24^ Twenty-eight per cent of registrars experience a major depressive episode during training, compared with the general population rate of 7% – 8%. According to Medscape’s 2019 report, 14% of physicians admitted to previous suicidal ideation and 1% had attempted suicide.^12^ Despite prevalence rates of 44% for burnout and 4% for clinical depression among physicians, only 13% were seeking professional help.

Considering these alarming figures, this study aimed to determine the rate and associated factors for burnout and depressive symptoms among doctors employed at Charlotte Maxeke Johannesburg Academic Hospital (CMJAH). We hypothesised that the burnout and depression rate would be elevated (± 50% of doctors) with an overlap of risk factors for each condition.

## Research methods and design

### Study design

This cross-sectional analysis of burnout and depression among doctors using the MBI–HSS for Medical Personnel and Patient-Health Questionnaire-9 (PHQ-9) was conducted between 22 November 2019 and 29 February 2020.

### Study setting

Charlotte Maxeke Johannesburg Academic Hospital is a state central level academic institution in Johannesburg, Gauteng, South Africa employing doctors across multiple disciplines and levels of training. All doctors in full-time employment (interns, medical officers, registrars and specialists) were approached to participate in the study.

### Measures

Once informed consent was obtained, paper-based questionnaires (MBI–HSS and PHQ-9) and a data collection form were self-reported and deposited into sealed, secure collection boxes that were collected by the primary investigator. Data were then recorded in an Excel spreadsheet. The analysed variables included sex, age, ethnicity, relationship status, job position, discipline, number of hours of overtime per week, number of hours remunerated work outside the public service (RWOPS) per week, academic and/or study time per week, hours per week spent on clerical work, psychiatric diagnosis, family history of psychiatric disorder, psychotherapy attendance, psychotropic medication and/or substance use in the past month and lifetime medication and/or substance use. An instrument was not scored if three or more items were missing; therefore, 327 MBI questionnaires and 335 PHQ-9 surveys were scored. Categories with *n* < 15 were not analysed.

### Data analysis

Burnout is defined as a high range score (≥ 27 points) on the EE subscale, a high range score (≥ 13 points) on the DP subscale and a low range score (≤ 31 points) on the PA subscale.^4^ Further definitions offered by Brenninkmeijer and VanYperen^25^ include the ‘Emotional Exhaustion + 1’ principle which states that a person can be diagnosed as being clinically burnt out with a high EE score plus either a high DP score or a low PA score. This definition encompasses a psychological state of sufficient severity to support a diagnosis of burnout.

For the purpose of this study, the ‘Emotional Exhaustion + 1’ principle^25^ was applied in defining burnout. Burnout was, therefore, defined as *high EE* (≥ 27 points) + *high DP* (≥ 13 points). The authors favoured this definition considering the participants different levels of experience. Furthermore, this was carried out noting that EE has been identified as the category that mostly indicates burnout. Individual measures of *high EE, high DP* and *low PA* (≤ 31 points) were also examined. A score of 27 and above in the EE subscale was considered high, 19–26 moderate and less than 19 low. A score of 10 and above in the DP subscale was considered high, 6–9 moderate and less than 6 low. A score of 40 and above in the PA subscale was considered high, 34–39 moderate and less than 34 low.^26^ The PHQ-9 has acceptable diagnostic properties at a range of cut-off scores (8–11).^27^ A score of ≥ 8 on the PHQ-9 was deemed indicative of depression.^27^

The association between risk factors and overall burnout and depressive symptoms was assessed by univariate binomial regression analysis. Factors with *p* < 0.20 were combined into a multivariable model, after examining each pair of variables for possible confounding using the chi-squared test (or Fisher’s exact test for 2×2 tables); a value of Cramer’s V (or the phi coefficient for Fisher’s exact test) > 0.50 was regarded as too strong an association to include both variables in the multivariable model. Non-significant variables were removed sequentially from the multivariable model to derive a final model. Data analysis was carried out using SAS version 9.4 for Windows. A 5% significance level was used.

### Ethical considerations

The study was approved by the University of the Witwatersrand, Human Research Ethics Committee. A distress protocol, detailing contact information of available resources for psychological and psychiatric care, was provided to all participants.

## Findings

Doctors in full-time employment at CMJAH over the study period were 694; 337 doctors for whom either the burnout or depression scale(s) could be scored participated in the study (overall response rate of 48.6%; margin of error 3.8%). The response rate for interns and registrars was significantly higher than other groups. A total of 58.8% (*n* = 198) of participants were female and 41.2% (*n* = 139) male (Table 1). Characteristics of the study participants are further described in Table 2. Variables were analysed for their association with burnout and depression (Tables 3 and 4).

**TABLE 1:** Response rate according to job position and discipline.

Category	Variable	Responses	Number of employees	Response (%)
Job position	Intern	40	63	63.5
	Medical officer	39	99	39.4
	Registrar	156	294	53.1
	Specialist	102	237	43.0
Discipline	Anaesthetics	34	78	43.6
	Emergency medicine	15	28	53.6
	Internal medicine	59	168	35.1
	Obstetrics and gynaecology	19	44	43.2
	Paediatrics	28	60	46.7
	Pathology	37	58	63.8
	Psychiatry	17	18	94.4
	Public health	4	7	57.1
	Radiology and nuclear medicine	18	45	40.0
	Surgery	106	188	56.4
Overall	-	337	694	48.6

**TABLE 2:** Characteristics of the study participants.

Characteristic	Overall (*N* = 337)	
	*n*	%
**Gender**		
Male	139	41.2
Female	198	58.8
**Age group (years)**		
20–29	82	24.3
30–39	183	54.3
40–49	54	16.0
50 or older	18	5.3
**Not in relationship**	93	27.6
**Self-reported ethnicity**		
African people	119	35.3
Caucasian people	115	34.1
Indian/Asian people	88	26.1
Other	15	4.5
**Job position**		
Intern	40	11.9
Medical officer	39	11.6
Registrar	156	46.3
Consultant	102	30.3
**Number of years since qualification (years)**		
0–5	82	24.3
6–8	84	24.9
9–12	81	24.0
13 or more	90	26.7
**Postgraduate qualification**	111	32.9
**Discipline**		
Surgery	106	31.5
Internal medicine	59	17.5
Pathology	37	11.0
Anaesthetics	34	10.1
Paediatrics	28	8.3
Obstetrics and gynaecology	19	5.6
Radiology or nuclear medicine	18	5.3
Psychiatry	17	5.0
Emergency medicine	15	4.5
Public health	4	1.2
**Full-time employment**	336	99.7
**Overtime (hours per week)**		
4 or less	22	6.6
5–12	43	12.8
13–20	89	26.4
More than 20	183	54.3
**Remunerative work outside the public service**	54	16.0
**Academic hours per week**		
Less than 10	221	65.6
10–30	106	31.5
More than 30	10	3.0
**Clerical hours per week**		
Less than 3	61	18.1
3–5	124	36.8
5–8	70	20.8
More than 8	82	24.3
**Any psychiatric diagnosis**	56	16.6
Before internship	19	5.6
After internship	37	11.0
**Psychiatric diagnosis**		
Depressive disorder	30	8.9
Anxiety disorder	30	8.9
Bipolar disorder	3	0.9
other	9	2.7
**Family history of psychiatric disorder**	87	25.8
**Psychotherapy**		
No	230	68.3
Yes – ongoing	23	6.8
Yes – previous	84	24.9
**Substance use in past month**		
Antidepressant and/or anxiolytic	50	14.8
Benzodiazepine	15	4.5
Stimulant	14	4.2
Illicit substance	10	3.0
Mood stabiliser	4	1.2
Antipsychotic	1	0.3
Other	4	1.2
None	264	78.3
**Previous substance use**		
Antidepressant and/or anxiolytic	32	9.5
Benzodiazepine	14	4.2
Stimulant	29	8.6
Illicit substance	11	3.3
Mood stabiliser	4	1.2
Antipsychotic	3	0.9
Other	3	0.9
None	267	79.2
**Burnout**		
High emotional exhaustion (EE ≥ 27)	199	60.9
High depersonalisation (DP ≥ 13)	196	59.9
Low personal accomplishment (PA ≤ 31)	181	55.4
Overall (EE ≥ 27 and DP ≥ 13)	151	46.2
Depressive symptoms (score of ≥ 8 on the PHQ-9)	180	53.7

EE, emotional exhaustion; DP, depersonalisation; PHQ, patient-health questionnaire; PA, personal accomplishment.

**TABLE 3:** Univariable analysis of risk factors for burnout and depressive symptoms.

Characteristic	High emotional exhaustion (EE ≥ 27)		High depersonalisation (DP ≥ 13)		Low personal accomplishment (PA ≤ 31)		Burnout		Depressive symptoms	
	RR	95% CI	RR	95% CI	RR	95% CI	RR	95% CI	RR	95% CI
**Female**	1.31†	1.08–1.59†	1.03	0.86–1.24	1.33	1.08–1.65	1.17	0.92–1.50	1.44	1.15–1.79†
**Age group (years)**										
20–29	1.00 *reference*	-	1.00 *reference*	-	1.00 *reference*	-	1.00 *reference*	-	1.00 *reference*	-
30–39	0.84	0.70–1.00	0.76	0.64–0.89†	1.00	0.80–1.25	0.69†	0.55–0.86†	0.86	0.70–1.06
40–49	0.72†	0.54–0.97†	0.53	0.37–0.75†	0.89	0.64–1.23	0.49†	0.31–0.75†	0.60†	0.42–0.87†
50 or older	0.26†	0.09–0.72†	0.48	0.25–0.91†	0.65	0.34–1.27	0.29†	0.10–0.81†	0.43†	0.20–0.92†
**Not in relationship**	1.03	0.85–1.25	1.08	0.89–1.31	1.15	0.94–1.41	1.01	0.78–1.32	1.03	0.83–1.28
**Self-reported ethnicity**										
African	1.00 *reference*	-	1.00 *reference*	-	1.00 *reference*	-	1.00 *reference*	-	1.00 *reference*	-
Caucasian	1.03	0.83–1.27	1.38†	1.11–1.71†	1.02	0.80–1.30	1.30	0.98–1.73	1.06	0.84–1.34
Indian/Asian	1.05	0.84–1.31	1.16	0.90–1.49	1.08	0.84–1.38	1.18	0.86–1.62	1.02	0.78–1.32
Other	1.08	0.71–1.65	1.26	0.82–1.94	1.20	0.79–1.84	1.26	0.71–2.22	0.95	0.55–1.65
**Job position**										
Intern	1.85†	1.40–2.43†	2.00†	1.52–2.64†	1.27	0.91–1.76	2.51†	1.76–3.57†	1.98†	1.42–2.76†
Medical officer	1.42†	1.01–1.99†	1.43†	1.00–2.04†	1.19	0.84–1.68	1.46	0.91–2.33	1.89†	1.34–2.66†
Registrar	1.54†	1.20–1.99†	1.61†	1.23–2.09†	1.24	0.97–1.59	1.66†	1.18–2.34†	1.66†	1.23–2.22†
Consultant	1.00 *reference*	-	1.00 *reference*	-	1.00 *reference*	-	1.00 *reference*	-	1.00 *reference*	-
**Number of years since qualification**										
0–5	1.54†	1.18–2.02†	1.98†	1.48–2.65†	1.25	0.93–1.68	2.20†	1.51–3.22†	1.83†	1.33–2.52†
6–8	1.47†	1.12–1.94†	1.77†	1.31–2.40†	1.31	0.98–1.75	1.73†	1.15–2.59†	1.72†	1.25–2.38†
9–12	1.31	0.98–1.75	1.45†	1.04–2.01†	1.28	0.95–1.72	1.64†	1.08–2.48†	1.46†	1.03–2.07†
13 or more	1.00 *reference*	-	1.00 *reference*	-	1.00 *reference*	-	1.00 *reference*	-	1.00 *reference*	-
**Postgraduate qualification**	0.64†	0.50–0.80†	0.61†	0.48–0.78†	0.90	0.72–1.11	0.55†	0.40–0.76†	0.60†	0.46–0.77†
**Discipline**										
Surgery	1.00 *reference*	-	1.00 *reference*	-	1.00 *reference*	-	1.00 *reference*	-	1.00 *reference*	-
Internal Medicine	0.83	0.60–1.13	0.99	0.75–1.30	1.03	0.73–1.45	0.93	0.64–1.36	0.91	0.64–1.29
Pathology	1.27	98–1.65	1.35†	1.05–1.73†	1.85†	1.43–2.40†	1.38	0.97–1.97	1.28	0.93–1.76
Anaesthetics	1.16	0.87–1.53	0.97	0.69–1.36	1.49†	1.09–2.04†	1.13	0.76–1.68	1.39†	1.03–1.89†
Paediatrics	1.30	0.99–1.71	1.04	0.73–1.49	1.06	0.67–1.68	1.08	0.68–1.72	1.10	0.74–1.64
O and G	1.26	0.92–1.72	1.19	0.84–1.68	1.39	0.93–2.09	1.31	0.84–2.02	1.49†	1.05–2.10†
Radiology or Nuclear Medicine	0.95	0.61–1.48	0.58	0.30–1.14	1.23	0.77–1.95	0.63	0.29–1.36	1.03	0.62–1.70
Psychiatry	0.80	0.47–1.37	0.92	0.57–1.48	1.17	0.71–1.92	0.53	0.22–1.28	1.09	0.67–1.78
Emergency Medicine	1.25	0.89–1.77	1.62†	1.31–2.01†	1.62†	1.12–2.34†	1.65†	1.14–2.40†	1.24	0.78–1.95
Public Health	0.43	0.08–2.35	0.87	0.32–2.35	1.66	0.91–3.03	0.56	0.10–3.12	1.03	0.38–2.80
**Overtime (hours per week)**										
4 or less	1.00 *reference*	-	1.00 *reference*	-	1.00 *reference*	-	1.00 *reference*	-	1.00 *reference*	-
5–12	1.08	0.56–2.09	0.67	0.39–1.16	1.08	0.69–1.68	0.92	0.43–1.99	1.15	0.60–2.22
13–20	1.67	0.94–2.94	1.21	0.82–1.81	1.05	0.70–1.58	1.48	0.78–2.82	1.42	0.79–2.56
More than 20	1.75†	1.00–3.04†	1.06	0.72–1.56	0.90	0.61–1.34	1.48	0.80–2.76	1.64	0.93–2.89
RWOPS	0.66†	0.48–0.91†	0.74†	0.55–0.99†	0.74	0.53–1.02	0.64†	0.42–0.97†	0.51†	0.33–0.77†
**Academic hours per week**										
Less than 10	1.00 *reference*	-	1.00 *reference*	-	1.00 *reference*	-	1.00 *reference*	-	1.00 *reference*	-
10–30	0.85	0.69–1.05	0.78†	0.63–0.97†	0.86	0.69–1.08	0.79	0.60–1.04	0.87	0.69–1.09
More than 30	1.26	0.91–1.75	0.61	0.29–1.32	0.86	0.46–1.61	0.80	0.37–1.74	0.71	0.33–1.52
**Clerical hours per week**										
Less than 3	1.00 *reference*	-	1.00 *reference*	-	1.00 *reference*	-	1.00 *reference*	-	1.00 *reference*	-
3–5	1.04	0.78–1.37	1.15	0.89–1.50	0.99	0.78–1.26	1.14	0.79–1.64	0.95	0.72–1.27
5–8	1.11	0.82–1.51	0.95	0.69–1.30	0.68†	0.48–0.95	1.12	0.75–1.68	0.87	0.62–1.22
More than 8	1.35†	1.03–1.77†	1.11	0.83–1.47	0.81	0.60–1.08	1.24	0.85–1.82	1.15	0.86–1.53
**Any psychiatric diagnosis**	1.48†	1.26–1.73†	1.22†	1.00–1.49†	1.43†	1.18–1.74†	1.47†	1.15–1.88†	1.70†	1.43–2.02†
Before internship	1.38†	1.05–1.80†	1.34†	1.03–1.75†	1.29	0.91–1.82	1.43	0.96–2.11	1.67†	1.37–2.04†
After internship	1.53†	1.29–1.81†	1.15	0.90–1.48	1.51†	1.22–1.86†	1.49†	1.13–1.97†	1.75†	-
**Psychiatric diagnosis**										
Depressive disorder	1.54†	1.32–1.81†	1.30†	1.04–1.63†	1.35†	1.05–1.73†	1.75†	1.37–2.23†	1.64†	1.35–1.99†
Anxiety disorder	1.55†	1.33–1.81†	1.38†	1.13–1.69†	1.37†	1.08–1.74†	1.60†	1.22–2.09†	1.62†	1.33–1.98†
**Family history of psychiatric disorder**	1.10	0.91–1.32	1.04	0.85–1.26	1.16	0.95–1.42	1.04	0.80–1.36	1.32†	1.08–1.61†
**Psychotherapy**										
No	-	-	-	-	-	-	-	-	-	-
Yes – ongoing	1.50†	1.20–1.87†	1.22	0.91–1.64	1.20	0.87–1.66	1.58†	1.13–2.21†	1.93†	1.56–2.39†
Yes – previous	1.28†	1.06–1.53†	1.15	0.95–1.40	1.02	0.81–1.29	1.32†	1.02–1.70†	1.53†	1.25–1.87†
**Substance use in past month**										
Antidepressant and/or anxiolytic	1.47†	1.25–1.73†	1.28†	1.05–1.55†	1.51†	1.25–1.82†	1.59†	1.25–2.01†	1.78†	1.51–2.10†
Benzodiazepine	1.33†	1.02–1.75†	1.48†	1.19–1.84†	1.48†	1.12–1.94	1.80†	1.35–2.38†	1.66†	1.33–2.08†
Stimulant	1.43†	1.14–1.81†	1.46†	1.15–1.84†	1.17	0.78–1.75	1.59†	1.11–2.26†	1.49†	1.11–2.00†
**Previous substance use**										
Antidepressant and/or anxiolytic	1.50†	1.27–1.77†	1.15	0.88–1.49	1.12	0.83–1.51	1.46†	1.09–1.95†	1.53†	1.23–1.89†
Benzodiazepine	1.31	0.98–1.74	1.33	1.00–1.77	0.90	0.53–1.53	1.42	0.94–2.13	1.49†	1.11–2.00†
Stimulant	0.96	0.70–1.32	1.23	0.97–1.57	1.07	0.77–1.47	1.13	0.78–1.65	1.32†	1.01–1.72†

EE, emotional exhaustion; DP, depersonalisation; RR, relative risk; CI, confidence interval.

† , Statistically significant RRs.

**TABLE 4:** Multivariable analysis of risk factors for burnout and depressive symptoms.

Characteristic	High emotional exhaustion (EE ≥ 27)		High depersonalisation (DP ≥ 13)		Low personal accomplishment (PA ≤ 31)		Burnout		Depressive symptoms	
	RR	95% CI	RR	95% CI	RR	95% CI	RR	95% CI	RR	95% CI
**Female**	-	-	-	-	1.31†	1.06–1.61†	-	-	1.18†	1.01–1.38†
**Self-reported ethnicity**										
African	1.00 *reference*	-	1.00 *reference*	-	1.00 *reference*	-	1.00 *reference*	-	1.00 *reference*	-
Caucasian	-	-	1.43	1.17–1.75†	-	-	1.32†	1.00–1.74†	-	-
Indian/Asian	-	-	1.21	0.96–1.53	-	-	1.16	0.84–1.60	-	-
Other	-	-	1.07	0.70–1.63	-	-	1.09	0.63–1.90	-	-
**Job position**										
Intern	1.45†	1.15–1.82†	1.86†	1.43–2.42†	-	-	2.23†	1.58–3.16†	1.43†	1.12–1.83†
Medical officer	1.20	0.95–1.52	1.45†	1.03–2.03†	-	-	1.43	0.91–2.23	1.34†	1.07–1.66†
Registrar	1.28†	1.08–1.53†	1.78†	1.38–2.30†	-	-	1.66†	1.19–2.32†	1.28†	1.06–1.55†
Consultant	1.00 *reference*	-	1.00 *reference*	-	1.00 *reference*	-	1.00 *reference*	-	1.00 *reference*	-
**Academic hours per week**										
Less than 10	1.00 *reference*	-	1.00 *reference*	-	1.00 *reference*	-	1.00 *reference*	-	1.00 *reference*	-
10–30	-	-	0.82†	0.67–0.99†	-	-	-	-	-	-
More than 30	-	-	0.71	0.33–1.53	-	-	-	-	-	-
**Any psychiatric diagnosis**	1.28†	1.11–1.47†	-	-	1.40†	1.16–1.69†	1.32†	1.05–1.67†	1.33†	1.16–1.52†

EE, emotional exhaustion; DP, depersonalisation; RR, relative risk; CI, confidence interval.

† , Statistically significant RRs.

### Burnout

The total number of doctors classified as experiencing burnout (high EE + high DP) was 151 (46.2%). Associations of statistical significance included age, job position, discipline, psychiatric diagnosis, the use of medication and/or substances and not performing RWOPS.

Risk factors for burnout can be divided into three sections:

- *Individual factors* – Female sex was not associated with overall burnout, but they had a higher risk of high DP and low PA; younger doctors (20–39 years) displayed a higher risk of burnout (87.4% of the study population) and high DP; Caucasians showed an increased risk of burnout and high DP.
- *Occupational factors* – Participants who screened positive for burnout constituted registrars (49.7%) and interns (19.9%); with interns, registrars and medical officers being at higher risk of high EE and high DP but not low PA. Burnout risk was increased, although not statistically significant, in emergency medicine (RR 1.65; CI 1.14–2.40, *p*-value 0.01) and pathology (RR 1.38; CI 0.97–1.97, *p*-value 0.07); high DP was associated with emergency medicine, pathology and obstetrics and gynaecology and low PA was associated with emergency medicine, public health, pathology and anaesthetics. Overtime was associated with high EE values but with an increased risk of overall burnout. Those who did RWOPS (mainly specialists) displayed lower risks of burnout (RR 0.64; CI 0.42–0.97, *p*-value 0.03) with a low/moderate EE and DP. Although academic and/or study hours were associated with high DP, it was associated with an increased risk of overall burnout. Longer clerical hours were associated with higher EE but not with an increased risk for burnout.
- *Psychiatric factors* – Psychiatric diagnosis was associated with an increased risk of burnout (RR 1.47; CI 1.15–1.38, *p*-value 0.00) and EE; in particular, 15.2% of these had a diagnosis made after internship (RR 1.49; CI 1.13–1.97, *p*-value 0.01) and 7.4% before internship (RR 1.43; CI 0.96–2.11, *p*-value 0.08). Significant psychiatric diagnoses included depressive disorders (75.9%) and anxiety disorders (70%). Psychiatric diagnosis was also associated with high DP if made before internship and low PA if made after internship. A total of 60.9% of doctors assessed as being burned-out had never attended psychotherapy; however, doctors who had previously engaged with or were currently engaging in psychotherapy were more likely to report feelings of burnout (RR 1.32; CI 1.02–1.70, p-value 0.03 and RR 1.58; CI 1.13–2.21, *p*-value 0.01, respectively). A significant association was observed between burnout and current use of antidepressants/anxiolytics (RR 1.59; CI 1.25–2.01, *p*-value 0.00), benzodiazepines (RR 1.80; CI 1.35–2.38, *p*-value < 0.0001) and stimulants (RR 1.59; CI 1.11–2.26, *p*-value 0.01) and previous use of antidepressants/anxiolytics (RR 1.46; CI 1.09–1.95, p-value 0.01) and benzodiazepines (RR 1.42; CI 0.94–2.13, p-value 0.09). Stimulant usage was unique because it increased during and/or after internship (shown in total overall burnout and high EE). High DP was associated with psychiatric diagnosis made before (but not after) internship and current (but not previous) medication usage. Low PA was associated with a psychiatric diagnosis made after internship and the current use of medications (antidepressants and benzodiazepines only).

There was no significant association between burnout and sex, relationship status, ethnicity, overtime hours per week, academic hours per week, clerical hours per week and family history of psychiatric disorder. Multivariate analysis revealed that burnout was associated with Caucasian race; intern and registrar positions and psychiatric diagnosis. Looking at the individual measures of burnout 60.9% (*n* = 199) of doctors scored high on EE, 59.9% (*n* = 196) scored high on DP; and 55.4% (*n* = 181) scored low on PA. Table 3 shows the variables examined and their relative risk of high EE, high DP and low PA.

### Depression

The number of participants who screened positive for depressive symptoms was 180 (53.73%) (Table 5). Significant association in the univariate analysis included female sex (RR 1.44; CI 1.15–1.79, *p*-value 0.00); younger age (highest in the 20–29-year-old group); being an intern (RR 1.98; CI 1.42–2.76, *p*-value < 0.0001), medical officer (RR 1.89; CI 1.34–2.66, *p*-value 0.00) or registrar (RR 1.66; CI 1.23–2.22, *p*-value 0.00); working in anaesthetics (RR 1.39; CI 1.03–1.89, *p*-value 0.03) and obstetrics and gynaecology (RR 1.49; CI 1.05–2.1, *p*-value 0.03); not performing RWOPS; psychiatric diagnosis (RR 1.7; CI 1.43–2.02, *p*-value < 0.001), particularly depressive (RR 1.64; CI 1.35–1.99, *p*-value < 0.001) or anxiety (RR 1.62; CI 1.33–1.98, *p*-value 0.001) disorders; psychiatric family history (RR 1.32; CI 1.08–1.61, *p*-value 0.01); past (RR 1.53; CI 1.25–1.87, *p*-value < 0.001) or present (RR 1.93; CI 1.56–2.39, *p*-value < 0.001) psychotherapy; and current or past use of antidepressants (RR 1.78; CI 1.51–2.1, *p*-value < 0.001 and RR 1.53; CI 1.23–1.89, *p*-value 0.00), benzodiazepines (RR 1.66; CI 1.33–2.08, *p*-value < 0.001 and RR 1.49; CI 1.11–2, *p*-value 0.01) or stimulants (RR 1.49; CI 1.11–2, *p*-value 0.01 and RR 1.32; CI 1.01–1.72, *p*-value 0.04). There was no significant association between depression and relationship status, ethnicity, overtime, and academic or clerical hours per week. Multivariate analysis revealed that depression was associated with female sex; interns, medical officer and registrar positions; and psychiatric diagnosis.

**TABLE 5:** Summary of significant associations with burnout, burnout subscales and depression.

Variable	Category	Depression	Burnout = high EE + high DP	High EE	High DP	Low PA
**Female sex**	-	x	-	x	-	x
**Younger age**	-	x	x	x	x	-
**Relationship status**	-	-	-	-	-	-
**Caucasian race**	-	-	x	-	x	-
**Job position**	Intern	x	x	x	x	-
	Medical officer	-	-	x	x	-
	Registrar	x	x	x	x	-
**Discipline**	Anaesthetics	x	-	-	-	x
	Emergency medicine	-	x	-	x	x
	Internal Medicine	-	-	-	-	-
	Obstetrics and gynaecology	x	-	-	x	-
	Paediatrics	-	-	-	-	-
	Pathology	-	-	-	x	x
	Psychiatry	-	-	-	-	-
	Public health	-	-	-	-	x
	Radiology and nuclear medicine	-	-	-	-	-
	Surgery	-	-	-	-	-
**More overtime hours**	-	-	x	x	x	-
**No RWOPS**	-	-	-	x	x	-
**Less academic hours**	-	-	-	-	-	-
**Clerical hours**	More	-	-	x	-	-
	Less	-	-	-	-	x
**Depression and/or anxiety diagnosis**	-	x	x	x	x	x
**Timing of diagnosis**	Before internship	x	x	x	x	-
	After internship	x	x	x	-	x
**Family history of psychiatric disorder**	-	x	-	-	-	-
**Psychotherapy**	Current	x	x	x	x	-
	Past	x	x	x	x	-
**Recent medication use**	Antidepressant and/or anxiolytic	x	x	x	x	x
	Benzodiazepine	x	x	x	x	x
	Stimulant	x	x	x	x	-
**Past medication use**	Antidepressant and/or anxiolytic	x	x	x	x	-
	Benzodiazepine	x	x	-	-	-
	Stimulant	x	-	-	-	-

RWOPS, remunerated work outside the public service; EE, emotional exhaustion; DP, depersonalisation PA, personal accomplishment.

## Discussion

### Burnout

Of the participants, 46.2% of doctors employed at CMJAH had burnout. This figure is in keeping with international rates^12^, but it is lower than rates found in South African studies.^8,9,21^ This could be attributed to a larger sample size in this study, with a broader range of disciplines and ranks of doctors assessed. We also utilised a definition of burnout that is a combination of the EE and DP subscales, whereas the majority of other SA studies considered individual subscales of burnout.

Those aged 20–39 were more likely to suffer burnout. Internship and registrarship in South Africa mostly commence within this age group and interns were found to be three times more likely to be burnt out than specialists. Common psychiatric disorders also tend to have their onset in this age group^28^; therefore, there may be an overlap in risk factors between burnout and mental illness. Factors contributing to results in this group may be organisational factors, clinical and clerical work demands and medical training insufficiently preparing doctors for the work environment, thus contributing to dysfunctional adjustment. Studies have indicated registrar training as a vulnerable time in which burnout is more likely to develop,^13,16,20,29^ possibly because of stressors of the academic training requirements, frequent rotations between training institutions and transitioning into more senior roles in the work environment.

Emergency medicine and pathology doctors were shown to be at a higher risk of burnout, whereas international data show that urology, neurology, internal medicine, surgery and anaesthetics had the highest burnout rates.^12^ The increased risk in emergency medicine in this setting may be because of the high output, community serviced by the hospital, crisis management service inherent to the discipline or because of specific departmental factors that need further exploration. Other studies have demonstrated that paramedic and emergency medicine personnel have high rates of burnout.^9,30,31^

Those who performed RWOPS were at a lower risk of burnout. In South Africa (SA), only specialists and medical officers are legally allowed to perform RWOPS. Although RWOPS performance increases work hours in this study, it was not found to increase the risk of burnout. This finding, however, may be confounded by a lower risk of burnout in specialists because of other factors, such as older age and less study time. One could also argue that while RWOPS increases time spent at work, remuneration for this work may decrease financial stress. Institutional factors may need to be examined in greater depth to account for this, for example, a lack of human and infrastructural resources may be more significant in the public versus the private healthcare sector.

Doctors with a psychiatric diagnosis (depression and anxiety) displayed a higher risk of burnout with the risk increasing during and after internship. This could be explained by various risk factors for mental illness during the period of internship, as outlined here. Whilst we observe the association between mental illness and burnout, it is difficult to statistically surmise causality from this finding, that is, if mental illness is an independent risk factor for burnout or if burnout predisposes one to develop mental illness.

Over 60% of doctors assessed as burnt out had never attended psychotherapy. There was a significant association between burnout and having past or present psychotherapy. Doctors may engage with psychotherapy for the management of depressive or anxiety disorders or to improve coping skills related to innate personality factors. It should be observed that the association strengthens with present psychotherapy. We can postulate that the current working environment exposes a need to engage in help-seeking behaviour, whether it is because of burnout itself or other psychiatric illness.

There was a significant association between burnout and the use of recent antidepressants and/or anxiolytics, benzodiazepines and stimulants, as well as for past use of antidepressants and/or anxiolytics and benzodiazepines. This may be because of either: (1) prescribed medication for a depressive and/or anxiety disorder or (2) self-medicating symptoms of either burnout or depression. The latter may be more likely as the association of benzodiazepine use and stimulant use versus antidepressant use is higher. Benzodiazepines are known for their anxiolytic and hypnotic properties, whereas stimulants may be utilised to manage symptoms of fatigue or improve alertness. This finding also implies that stimulant use began or increased after internship. This is an interesting finding, as the number of doctors who, according to our survey, had a diagnosis of ‘other’ was 9 out of 337 (2.7%) (Attention deficit hyperactive disorder [ADHD], the most common condition, for which stimulants such as methylphenidate are prescribed, would be included here).

There was no significant association between burnout and sex, relationship status, ethnicity, overtime per week, academic hours per week, clerical hours per week and family history of psychiatric disorder. Some of these findings differ from what has been reported in the literature; it has been reported that females, clerical work and academic or study time have also been cited as risk factors of burnout. This study showed that females are at increased risk of depressive symptoms (in keeping with accepted risk factors for depression^32,33^) but not burnout. This implies that, although burnout and depression may have some common risk factors, sex may not be one of them in this population and it is in keeping with findings from a 2012 systematic review.^13^

Multivariate analysis revealed that burnout was associated with Caucasian race. There are little data regarding ethnicity as a risk factor for burnout. South Africa has a unique history regarding racial divides, but it is uncertain whether this is a factor that needs to be considered in the light of this finding.

### Individual measures of burnout

Three different measures of burnout were analysed individually to determine if particular associations differed from the definition of burnout utilised here (EE + DP). A total of 60.9% of doctors scored high on EE; 59.9% scored high on DP and 55.4% scored low on PA. Global rates of EE range between 43% and 80%, which was comparable to what we found.^34^ Global rates for high DP are 35% – 61% (similar to our rates) and 32% – 44% for low PA (lower than our rates). Longer overtime and clerical hours were high EE risk factors, which could be explained by increased emotional and physical fatigue. Having a psychiatric diagnosis, the use of medications and engagement in past or present psychotherapy were all associated with high EE. These three categories are closely related to each other. Having a pre-existing mental illness may be a risk factor for or a result of high EE. Symptoms of depression and anxiety may be the same as those found in EE. We hypothesise that medications could be used by doctors to either manage EE or a mental illness. Interestingly, EE was associated more with psychiatric diagnosis after internship, further enmeshing the symptomatology between these two parameters.

Depersonalisation represents the interpersonal element of burnout and it appears that certain disciplines (such as emergency medicine and obstetrics and gynaecology) may be particularly vulnerable in terms of this aspect of burnout but not EE or PA. High levels of DP were found in Caucasians and it is uncertain why this may be a factor in our setting. Those who performed less than 10 h per week of academic duties had the highest risk; this may be linked to interns, medical officers and specialists when compared with registrars (who are enrolled in a training programme), engaging in less academic activities. It also implies that high DP is associated more with other risk factors rather than the academic aspects of medicine. Psychiatric diagnosis made *before* internship was associated with increased risk of high DP; it can, therefore, be surmised that pre-existing mental illness will dictate higher levels of DP. It is the only subscale that shows this particular risk pattern, as PA was only associated with increased risk after internship, and EE was associated with an increased risk both before and after internship (the risk being higher after internship).

Females were found to be at higher risk of low PA. One explanation is that females often face more discrimination in the workplace compared with their male counterparts.^35,36^ The disciplines of pathology, public health, emergency medicine and anaesthetics were found to have low PA scores and this will require exploration in a larger scale study. While the discipline of emergency medicine was significantly associated with high overall burnout, anaesthetics was associated with higher depressive symptoms.

### Depression

Doctors have higher rates of depression and suicide when compared with the general population.^14,37^ Of the participants, 53.31% at CMJAH screened positive for depressive symptoms higher than the reported rates in previous studies (30% – 40.7%).^21,22^ A systematic review on registrars indicated a prevalence of 1% – 56% for depression.^37^ This may be linked to using a cut-off score of eight on the PHQ-9 in order not to miss milder forms of depression, which may have been understated in other clinician populations.

In terms of individual and psychiatric factors, our findings are in keeping with most known risk factors for depression viz. female sex, age of onset (20–30), a past episode of depression and positive family history.^38^ Relationship status was, however, not associated with depressive symptoms in this study; this differs from the literature with those who are single, widowed or divorced are at a higher risk of depression.^39^ Ethnicity was also shown not to be a risk factor, which is also in keeping with other findings in the SA context.^40,41^

While there are global variances in rates of depression between medical disciplines, in this study, the disciplines of anaesthetics and obstetrics and gynaecology had a significantly elevated risk for depressive symptoms but not for overall burnout. Anaesthetists are more likely to complete suicidal acts and more likely to die by substance-related suicide when compared with the general population.^42^ This concerning finding warrants further investigation and application of appropriate preventative and other interventions.

The current use of medications such as antidepressants, benzodiazepines and stimulants was associated with depression and anxiety. While we can conclude that the use of antidepressants is likely to be for the treatment of depression, we cannot conclusively say the same for the latter two groups of medication. We know that doctors are less likely to seek medical help and are more likely to self-medicate.^12^ We also know that that long-term benzodiazepine use may be linked to depressive symptoms.^43,44,45^

The bidirectional relationship between burnout and depression makes it difficult to determine causality over the course of both conditions. A recent systematic review and meta-analysis concluded that job strain may precipitate clinical depression.^46^ Although there is an overlap of both symptoms and risk factors, it appears, from our findings, that unique risk factors for depression amongst doctors are female sex, family history of depression and working in the fields of anaesthetics and obstetrics and gynaecology. For burnout, the only standalone factors of note are Caucasian race and working in the discipline of emergency medicine. If one examines the individual burnout subscales, other factors may contribute. Younger age is a common risk factor for both conditions and junior doctors were more likely to be both depressed and burnt out.

## Conclusion and recommendations

High burnout and depression rates were found in this study. There appears to be a bidirectional relationship between the two conditions. Although they have shared risk factors, specific risk factors may be determined for each in this population and confirmed with a longitudinal study looking at the causality between burnout and mental illness. Further studies with semi-structured interviews within the institution may determine organisational and departmental factors, impacting the high rate of burnout and depression, especially within disciplines with significant findings, for example, anaesthetics, emergency medicine and obstetrics and gynaecology.

While this study briefly reflected on the use of medications, the study population is unique because doctors have easier access to medication when compared with the general population. In this regard, it may be interesting to note how many of the physicians self-prescribed medication to manage burnout and depressive symptoms.

It is important to determine the incidence of burnout and factors associated with it to tailor interventions appropriately.^47^ Organisation-based interventions target workload reductions, enhancing teamwork, structured schedules and supervising job demands.^47^ Individual-based interventions involve psychological therapies, such as cognitive behavioural therapy and mindfulness, as well as strategies at improving coping and communication.^48^ There is an emphasis on convening physicians in social settings (a shared space), improving communication and decreasing stress. However, there are currently no definitive guidelines or interventions that are considered applicable in all settings for all professionals. Given the prevalence and risk burnout poses to both physicians and patients, the development of guidelines on how to manage this condition would be useful. Further research in this regard should be encouraged and should focus on establishing a cohesive strategy between individual and organisation interventions to decrease burnout rates.

### Limitations

There are several limitations to this study. Firstly, self-reported questionnaires were utilised and these are dependent on participants, who may over- or under-report symptoms. The response rate for interns and registrars was higher, which may indicate responder bias, showing that these groups were more likely to screen positive for burnout and depression. Additionally, findings at a tertiary-level academic institution in an urban setting in South Africa may not be generalisable to other areas of healthcare. This study utilised the PHQ-9, which is a screening tool, and therefore, a formal clinician review would be needed to make a diagnosis of depression. Finally, this study determined associations between risk and certain variables, and therefore, we cannot comment on causality.

## References

[1] Freudenberger HJ. Staff burnout. J Soc Issues. 1974;30(1):159–165. 10.1111/j.1540-4560.1974.tb00706.x

[2] Maslach C, Schaufeli WB, Leiter M. Job burnout. Annu Rev Psychol. 2001;52:397–422. 10.1146/annurev.psych.52.1.397 11148311

[3] Maslach C, Jackson SE. The measurement of experienced burnout. J Organ Behav. 1981;2(2):99–113. 10.1002/job.4030020205

[4] Schaufeli WB, Enzmann D. The burnout companion to study and practice: A critical analysis. London: Taylor & Francis; 1998.

[5] Stodel JM, Stewart-Smith A. The influence of burnout on skills retention of junior doctors at Red Cross War Memorial Children’s Hospital: A case study. S Afr Med J. 2011;101(2):115–118. 10.7196/SAMJ.4431 21678738

[6] Van der Walt N, Scribante J, Perrie H. Burnout among anaesthetists in South Africa. S Afr J Anaesth Analg. 2015;21(6):27–30. 10.1080/22201181.2015.1102798

[7] Sirsawy U, Steinberg WJ. Levels of burnout among registrars and medical officers working at Bloemfontein public healthcare facilities in 2013. S Afr J Fam Physicians. 2016;58(6):213–218. 10.1080/20786190.2016.1198088

[8] Liebenberg AR, Coetzee JF, Conradie HH, et al. Burnout among rural hospital doctors in the Western Cape: Comparison with previous South African studies. Afr J Prim Health Care Fam Med. 2018;10(1):1–7. 10.4102/phcfm.v10i1.1568 PMC6018597 29943596

[9] Rajan S, Engelbrecht A. A cross-sectional survey of burnout amongst doctors in a cohort of public sector emergency centres in Gauteng, South Africa. Afr J Emerg Med. 2018;8(13):95–99. 10.1016/j.afjem.2018.04.001 30456156 PMC6223592

[10] Zeijlemaker C, Moosa S. The prevalence of burnout among registrars in the School of Clinical Medicine at the University of the Witwatersrand, Johannesburg, South Africa. S Afr Med J. 2019;109(9):668–672. 10.7196/SAMJ.2019.v109i9.13667 31635592

[11] Coetzee JF, Kluyts H. Burnout and areas of work-life among anaesthetists in South Africa Part 1: Burnout. S Afr J Anaesth Analg. 2020; 26(2):73–82. 10.36303/SAJAA.2020.26.2.2358

[12] Medscape. Medscape National Physician Burnout, depression and suicide report [document on the Internet]. 2019 [cited 2020 Nov 21]. Available from: https://www.medscape.com/slideshow/2019-lifestyle-burnout-depression-6011056.

[13] Bria M, Baban A, Dumitrascu D. Systematic review of burnout risk factors among European healthcare professionals. Cogn Brain Behav. 2012;10(3):423–452.

[14] Patel RS, Bachu R, Adikey A, et al. Factors related to physician burnout and its consequences: A review. Behav Sci. 2018;8(11):98. 10.3390/bs8110098 30366419 PMC6262585

[15] Maslach C, Leiter MP. Understanding the burnout experience: Recent research and its implications for psychiatry. World Psychiatry. 2016;15(2):103–111. 10.1002/wps.20311 27265691 PMC4911781

[16] Amoafo E, Hanbali N, Patel A, et al. What are the significant factors associated with burnout in doctors. Occup Med. 2015;65(2):117–121. 10.1093/occmed/kqu144 25324485

[17] Salvagioni DAJ, Melanda FN, Mesas AE, et al. Physical, psychological and occupational consequences of job burnout: A systematic review of prospective studies. PLoS One. 2017;12(10):e0185781. 10.1371/journal.pone.0185781 28977041 PMC5627926

[18] Schonfeld IS, Verkuilen J. Inquiry into the correlation between burnout and depression. J Occup Health Psychol. 2019;24(6):603–616. 10.1037/ocp0000151 30945922

[19] Schonfeld IS, Bianchi R. Burnout and depression: Two entities or one. J Clin Psychol. 2016;72(1):22–37. 10.1002/jclp.22229 26451877

[20] Douglas A, Mata DA, Marco A, et al. Prevalence of depression and depressive symptoms among resident physicians: A systematic review and meta-analysis. J Am Med Assoc. 2015;314(22):2373–2383. 10.1001/jama.2015.15845 PMC4866499 26647259

[21] Rossouw L, Seedat S, Emsley RA, et al. The prevalence of burnout and depression in medical doctors working in the Cape Town Metropolitan Municipality community healthcare clinics and district hospitals of the Provincial Government of the Western Cape: A cross-sectional study. S Afr Fam Pract. 2013;55(6):567–573. 10.1080/20786204.2013.10874418

[22] Naidu K, Torline JR, Henry M, et al. Depressive symptoms and associated factors in medical interns at a tertiary hospital. S Afr J Psychiatr. 2019;25(1):1–8. 10.4102/sajpsychiatry.v25i0.1322 PMC6620542 31308973

[23] Naidoo T, Tomita A, Paruk A. Burnout, anxiety and depression risk in medical doctors working in KwaZulu-Natal Province, South Africa: Evidence from a multi-site study of resource-constrained government hospitals in a generalised HIV epidemic setting. PLoS One. 2020;15(10):e0239753. 10.1371/journal.pone.0239753 33052921 PMC7556533

[24] American Foundation for Suicide Prevention (n.d.). Ten facts about physician suicide and mental health [homepage on the Internet]. No date [cited 2020 Nov 06]. Available from: https://afsp.org/our-work/education/healthcare-professional-burnout-depression-suicide-prevention/#section2.

[25] Brenninkmeijer V, VanYperen N. How to conduct research on burnout: Advantages and disadvantages of a unidimensional approach in burnout research. Occup Environ Medicine. 2003;60(suppl 1):i16–20. 10.1136/oem.60.suppl_1.i16 PMC1765718 12782742

[26] Doulougeri K, Georganta K, Montgomery A. ‘Diagnosing’ burnout among healthcare professionals: Can we find consensus? Cogent Med. 2016;3(1):1. 10.1080/2331205X.2016.1237605

[27] Manea L, Gilbody S, McMillan D. Optimal cut-off score for diagnosing depression with the Patient Health Questionnaire (PHQ-9): A meta-analysis. CMAJ. 2012;184(3):E191–E196. 10.1503/cmaj.110829 22184363 PMC3281183

[28] Thomas NK. Resident burnout. J Am Med Assoc 2004;292(23):2880–2889. 10.1001/jama.292.23.2880 15598920

[29] Arora M, Asha S, Chinnappa J. Review article: Burnout in emergency medicine physicians. Emerg Med Australas. 2013;25(6):491–495. 10.1111/1742-6723.12135 24118838

[30] Kessler RC, Berglund P, Dember O, et al. Lifetime prevalence and age-of-onset distributions of DSM-IV disorders in the National Comorbidity Survey Replication. Arch Gen Psychiatry. 2005;62(6):593–602. 10.1001/archpsyc.62.6.593 15939837

[31] Strassen W, Van Nugteren B, Stein C. Burnout among advanced life support paramedics in Johannesburg, South Africa. Emerg Med J. 2013;30(4):331–334. 10.1136/emermed-2011-200920 22505296

[32] Gutiérrez-Rojas L, Porras-Segovia A, Dunne H, et al. Prevalence and correlates of major depressive disorder: A systematic review. Braz J Psychiatry 2020;42(6):657–672. 10.1590/1516-4446-2020-0650 32756809 PMC7678895

[33] Kwong ASF, López-López JA, Hammerton G, et al. Genetic and environmental risk factors associated with the trajectory of depressive symptoms from adolescence to young adulthood. JAMA Netw Open 2019;2(6):e196587. 10.1001/jamanetworkopen.2019.6587 31251383 PMC6604106

[34] Kumar S. Burnout and doctors: Prevalence, prevention and intervention. Healthcare (Basel) 2016;4(3):37. 10.3390/healthcare4030037 27417625 PMC5041038

[35] Coombs AAT, King RK. Workplace discrimination: Experiences of practising physicians. J Natl Med Assoc 2005;97(4):467–477. 15868767 PMC2568696

[36] Smith DT, Jacobson CK. Racial and gender disparities in the physician assistant profession. Health Serv Res 2016;51(3):892–909. 10.1111/1475-6773.12358 26368316 PMC4874816

[37] Joules N, Williams DM, Thompson A. Depression in resident physicians: A systematic review. Open J Depress 2014;3(3):89–100. 10.4236/ojd.2014.33013

[38] Köhler CA, Evangelou E, Stubbs B, et al. Mapping risk factors for depression across the lifespan. J Psychiatr Res 2018;103:189–207. 10.1016/j.jpsychires.2018.05.020 29886003

[39] Gariépy G, Honkaniemi H, Quesnel-Vallée A. Social support and protection from depression: systematic review of current findings in Western countries. British J Psychiatry 2016;209(4):284–293. 10.1192/bjp.bp.115.169094 27445355

[40] Bantjies J, Lochner C, Saal W, et al. Prevalence and sociodemographic correlates of common mental disorders among first-year university students in post-apartheid South Africa: Implications for a public mental health approach to student wellness. BMC Public Health 2019;19(922): 10.1186/s12889-019-7218-y PMC6617861 31291925

[41] National Mental Health Policy Framework and Strategic Plan 2013–2020 [homepage on the Internet]. [cited 2020 Sep 06]. Available from: https://health-e.org.za/2014/10/23/policy-national-mental-health-policy-framework-strategic-plan-2013-2020/.

[42] Swanson SP, Roberts LJ, Chapman MD. Are anaesthetists prone to suicide? A review of rates and risk factors. Anaesth Intensive Care 2003;31(4):434–445. 10.1177/0310057X0303100413 12973968

[43] Smith BD, Salzman C. Do benzodiazepines cause depression. Hosp Community Psychiatry 1991;42(11):1101–1102. 10.1176/ps.42.11.1101 1743635

[44] Longo L, Johnson B. Addiction: Part I. Benzodiazepines - side effects, abuse risk and alternatives. Am Fam Physician 2000;1(61):2121–2128. 10779253

[45] Diagnostic and statistical manual of mental disorders, 5th ed. American Psychiatric Association: Arlington, VA; 2013.

[46] Madsen IEH, Nyberg ST, Magnusson Hanson LL, et al. Job strain as a risk factor for clinical depression: Systematic review and meta-analysis with additional individual participant data. Psychol Med 2017;47(8):1342–1356. 10.1017/S003329171600355X 28122650 PMC5471831

[47] Awa WL, Plaumann M, Walter U. Burnout prevention: A review of intervention programs. Patient Educ Couns 2010;78(2):184–190. 10.1016/j.pec.2009.04.008 19467822

[48] Wiederhold BK, Cipresso P, Pizzioli D, et al. Intervention for physician burnout: A systematic review. Open Med 2018;13:253–263. 10.1515/med-2018-0039 PMC6034099 29992189

